# Effects of imatinib mesylate on cutaneous neurofibromas associated with neurofibromatosis type 1

**DOI:** 10.1002/ccr3.3071

**Published:** 2020-06-26

**Authors:** Ken‐ichi Yasuda, Yoshimasa Nobeyama, Takaoki Ishiji, Arihito Ota, Akihiko Asahina

**Affiliations:** ^1^ Department of Dermatology The Jikei University School of Medicine Tokyo Japan

**Keywords:** cutaneous neurofibroma, imatinib mesylate, neurofibromatosis type 1, NF1, paradoxical reaction, RASopathy

## Abstract

Imatinib mesylate seemed to inhibit development of cutaneous neurofibromas (c‐NFs) and promote growth of pre‐existing c‐NFs in our neurofibromatosis type 1 case. This report potentially provides new findings in the effects of imatinib mesylate.

## Graphical abstract

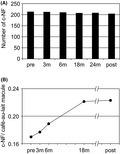

## 1 INTRODUCTION AND AIM

RASopathy is a comprehensive concept covering multiple genetic syndromes caused by germline mutations of the genes that encode the molecules associated with the RAS/mitogen‐activated protein kinase (MAPK) pathway. RASopathy includes Noonan syndrome, Costello syndrome, Legius syndrome, and neurofibromatosis type 1 (NF1) NF1 is an autosomal dominant disease present with various symptoms and signs, including café‐au‐lait spots, axillary freckling, cutaneous neurofibromas (c‐NFs), and plexiform neurofibromas. The disease is mainly caused by mutation of *NF1* gene encoding neurofibromin, which is a GTPase‐activating protein that negatively regulates the RAS/MAPK pathway by accelerating the hydrolysis of RAS‐bound GTP.

Gastrointestinal stromal tumor (GIST) is a mesenchymal tumor originating from the digestive tract. The tumor is generally positive for c‐Kit protein and is primarily caused by activating mutation of KIT proto‐oncogene, receptor tyrosine kinase gene (*c‐Kit*), or platelet‐derived growth factor receptor alpha gene (*PDGFRA*). Previous studies reported that GIST occurs in 5%‐29% of NF1 patients, and GISTs associated with NF1 (NF1‐GISTs) account for 1%‐2% of primary sporadic GISTs.^1^ NF1‐GISTs also commonly express c‐Kit protein, but they rarely have *c‐Kit* mutation.^1^

Imatinib mesylate has been reported to inhibit the growth of plexiform neurofibromas in NF1 by negatively regulating at least phospho‐signaling cascades.^2^ However, whether imatinib mesylate has any effect on c‐NFs in NF1 has not yet been elucidated. A case of NF1 with c‐Kit‐expressing GIST, who was unexpectedly treated by imatinib mesylate in another facility, is presented. The aim of this study was to elucidate the effects of imatinib mesylate on c‐NFs through analyses of the number and size of c‐NFs in this patient.

## 2 CASE AND METHODS

A 32‐year‐old Japanese man with NF1 was referred to us with a two‐decade history of numerous soft cutaneous nodules. Physical examination showed eight café‐au‐lait macules measuring >1.5 cm in diameter and numerous c‐NFs. The patient was then diagnosed as having NF1 according to the diagnostic criteria of the National Institutes of Health consensus development conference 1988. Magnetic resonance imaging examination unexpectedly detected an intra‐abdominal tumor, and it was resected surgically. Histopathological examination showed that the tumor was a c‐Kit‐expressing GIST. Then, imatinib mesylate 300‐400 mg/d was administered for two years in another facility. While imatinib mesylate was being administered, the patient underwent resection of several c‐NFs twice in our department. The GIST has not shown recurrence 12 months after termination of imatinib mesylate.

**Figure 1:**
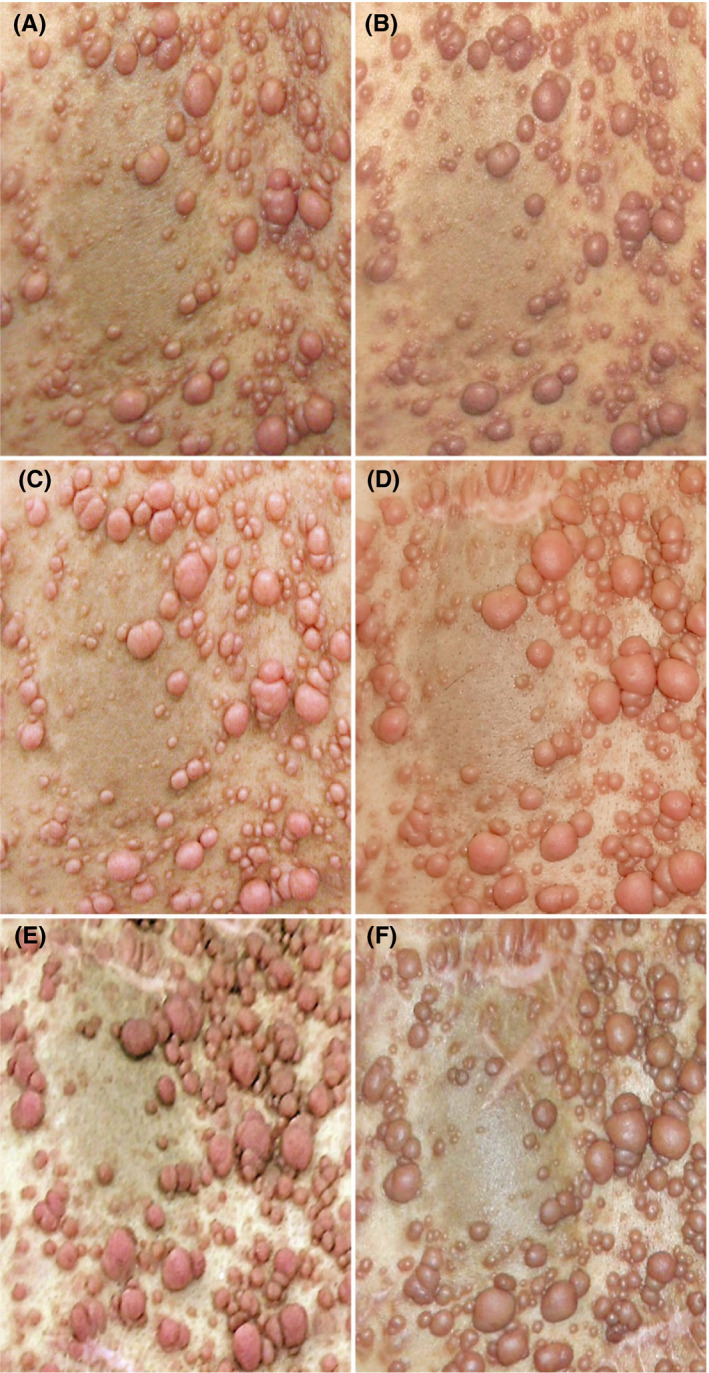
Photographs before, during, and after administration of imatinib mesylate. Photographs are arranged in the same range including the café‐au‐lait macule assigned as a marker on his back. A, Photograph just before administration. B, Photograph at 3 mo after initiation of administration. C, Photograph at 6 mo after initiation of administration. D, Photograph at 18 mo after initiation of administration. A new scar due to the resection of several c‐NFs is evident. E, Photograph at 24 mo after initiation of administration. F, Photograph at 12 mo after termination of administration. A new scar due to the resection of several c‐NFs is evident

The cutaneous manifestations were recorded by photographs just before imatinib mesylate administration, at 3, 6, 18, 24 months after initiation of its administration, and 12 months after its termination (Figure 1). A particular area on the back was defined as a location for analysis, because the café‐au‐lait macule in that area was considered to be suitable as a location and size marker. The patient underwent resection of several c‐NFs between 6 and 18 months after initiation of imatinib mesylate administration, and between 24 months after its initiation and 12 months after its termination. The photographic images in that area were analyzed chronologically as follows: (a) The number of all c‐NFs was counted; and (b) the longest diameter of each of 10 representative c‐NFs and the longest diameter perpendicular to the maximum diameter of the café‐au‐lait macule assigned as a marker were measured. Because the photograph taken at 24 months was obliquely shot, it was used for counting, but not for measurement.

## 3 RESULTS

### 3.1 Number of c‐NFs decreased slightly

**Figure 2:**
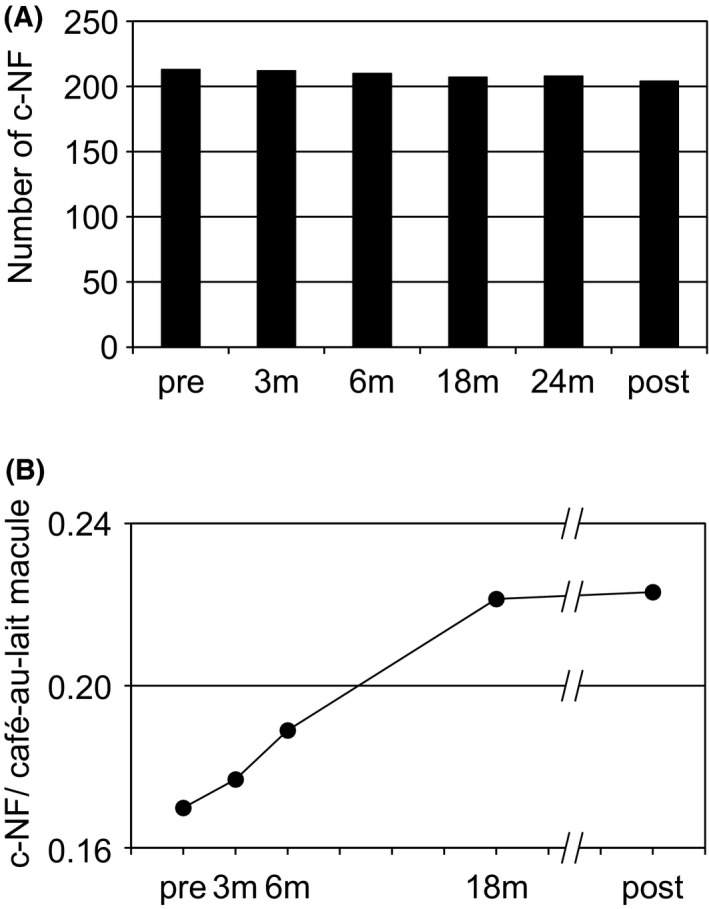
Changes in number and size of c‐NFs. A, Number of c‐NFs at each examination point. Vertical and horizontal axes indicate the number of c‐NFs and examination time points, respectively. Pre, 3, 6, 18, 24 mo, and post indicate each examination time point just before imatinib mesylate administration, at 3, 6, 18, and 24 mo after initiation of administration, and 12 mo after termination of administration, respectively. B, Size ratio of the representative c‐NFs to the café‐au‐lait macule assigned as a marker at each examination time point. The size ratio is defined as the average of the 10 values calculated from the longest diameter of each of the 10 representative c‐NFs divided by the longest diameter perpendicular to the maximum diameter of the café‐au‐lait macule assigned as a marker. Vertical and horizontal axes indicate the size ratio and examination time points, respectively. Pre, 3, 6, 18 mo, and post indicate each examination time point just before imatinib mesylate administration, at 3, 6, 18 mo after initiation of administration, and 12 mo after termination of administration, respectively

The number of c‐NFs within the defined area on the back was 213 just before administration of imatinib mesylate, 212 at 3 months, 210 at 6 months, 207 at 18 months, 208 at 24 months after its initiation, and finally, 204 at 12 months after its termination (Figure 2A).

### 3.2 Size of c‐NFs increased

For each of the 10 representative c‐NFs, the maximum diameter was divided by the longest diameter perpendicular to the maximum diameter of the representative café‐au‐lait macule. The average values and their time transition were shown in Table 1 and Figure 2B, respectively. The statistical analyses (Friedman test) revealed significant differences among the values during its initiation to 18 months after its initiation, and no significant difference between 18 months after its initiation and 12 months after its termination (Table 2).

**Table 1:** Data of size ratio of the representative c‐NFs to the café‐au‐lait macule

		Pre	3 mo	6 mo	18 mo	Post
c‐NF	#1	0.210	0.235	0.229	0.277	0.289
c‐NF	#2	0.210	0.208	0.238	0.262	0.275
c‐NF	#3	0.228	0.231	0.247	0.272	0.260
c‐NF	#4	0.164	0.186	0.185	0.214	0.216
c‐NF	#5	0.164	0.154	0.203	0.252	0.211
c‐NF	#6	0.110	0.113	0.123	0.155	0.172
c‐NF	#7	0.183	0.190	0.181	0.233	0.235
c‐NF	#8	0.137	0.145	0.176	0.228	0.240
c‐NF	#9	0.146	0.154	0.150	0.155	0.157
c‐NF	#10	0.146	0.154	0.159	0.165	0.176
Average		0.170	0.177	0.189	0.221	0.223

Note

Pre, just before starting imatinib mesylate. 3 mo, at 3 mo after imatinib mesylate initiation. 6 mo, at 6 mo after imatinib mesylate initiation. 18 mo, at 18 mo after imatinib mesylate initiation. Post, at 12 mo after imatinib mesylate termination.

**Table 2:** *P*‐value in comparison of c‐NF size between each time point

Time point	*P*‐value (Friedman test)
Pre vs 3 mo	1.000
Pre vs 6 mo	.897
Pre vs 18 mo	<.001
Pre vs post	<.001
3 mo vs 6 mo	1.000
3 mo vs 18 mo	.047
3 mo vs post	.002
6 mo vs 18 mo	.162
6 mo vs post	.011
18 mo vs post	1.000

Note

Pre, just before starting imatinib mesylate. 3 mo, at 3 mo after imatinib mesylate initiation. 6 mo, at 6 mo after imatinib mesylate initiation. 18 mo, at 18 mo after imatinib mesylate initiation. Post, at 12 mo after imatinib mesylate termination.

## 4 DISCUSSION

The results of the present study suggest that imatinib mesylate has inhibitory effects on the development of c‐NFs and growth‐promoting effects on pre‐existing c‐NFs in NF1, although only a single case was examined.

NF1‐GISTs have distinct phenotypes compared with common GISTs as follows: young age onset, distal localization, small size, and low mitotic rate. In addition, although c‐Kit protein is commonly overexpressed in NF1‐GISTs, mutation of *c‐Kit* or *PDGFRA* is only rarely observed.^3^ In that context, imatinib mesylate is supposed to be generally ineffective for NF1‐GISTs.^3^ The present patient was given imatinib mesylate in another facility without being checked for *c‐Kit* mutation and without considering the implications of the associated NF1. Therefore, this observational study was based on a rare and unexpected opportunity.

The number of c‐NFs in the present patient decreased slightly during the observation period. Considering that multiple c‐NFs were surgically resected during the observation period, the total number of c‐NFs may indeed have remained almost unchanged. The results thus suggest that imatinib mesylate inhibits the development of de novo c‐NFs. Jouhilahti et al suggested that the development of c‐NFs requires recruitment of multipotent NF1^+/−^ precursor cells,^4^ which have functional neurofibromin produced from monoallelic *NF1* gene. Both negative regulation of tyrosine kinase receptors by imatinib mesylate and negative regulation of RAS by functional neurofibromin may potentially contribute to suppression of the development of c‐NFs from multipotent NF1^+/−^ precursor cells.

The size of c‐NFs in the present patient was significantly increased during the administration of imatinib mesylate and became almost unchanged after the termination of its administration. These results suggest that imatinib mesylate has growth‐promoting effects on pre‐existing c‐NFs. As a possible mechanism, because of the loss of function of neurofibromin to inhibit RAS, imatinib mesylate may somehow act on tyrosine kinase receptors in *NF1* ^‐/‐^ cells in pre‐existing c‐NFs, resulting in activation of the RAS/MAPK signaling pathway. This phenomenon should be referred to as a "paradoxical reaction" of imatinib mesylate to c‐NFs in NF1. Brosseau et al suggested potential differences between c‐NFs and plexiform neurofibromas in terms of cellular origin, *NF1* genotype, cellular composition, and tumor microenvironment.^5^ Therefore, the difference in the effect of imatinib mesylate between c‐NFs and plexiform neurofibromas may not be unexpected, although the underlying mechanisms responsible for the difference should be investigated further.

The main limitation of the study is that only a single case was analyzed. Other limitations are that the observation period was not long enough to assess the number of c‐NFs after the termination of imatinib mesylate administration, and that several c‐NFs in the defined area for analysis were surgically resected in that period; these factors may have hampered confirmation of the increase in the number of c‐NFs after the termination of imatinib mesylate administration.

In conclusion, the present study suggests that imatinib mesylate has inhibitory effects on the development of c‐NFs and growth‐promoting effects on pre‐existing c‐NFs.

## CONFLICT OF INTEREST

The authors have no conflicts of interest to declare.

## AUTHOR CONTRIBUTIONS

KY: contributed to data curation and resources. YN: contributed to conceptualization and project administration. TI and AO: involved in resources and supervision. AA: contributed to review and supervision.
